# Musculoskeletal pain among male faculty members of the College of Medicine and College of Dentistry

**DOI:** 10.1097/MD.0000000000026176

**Published:** 2021-05-28

**Authors:** Osama R. Aldhafian, Faisal A. Alsamari, Naif A. Alshahrani, Mohammed N. Alajmi, Abdulelah M. Alotaibi, Naif Bin Nwihadh, Ayman K. Saleh

**Affiliations:** a Department of Surgery, College of Medicine, Prince Sattam Bin Abdulaziz University, Al-Kharj, Kingdom of Saudi Arabia; b Department of Orthopaedics, faculty of Medicine for girls, Alazhar university, Cairo, Egypt.

**Keywords:** College of Dentistry, College of Medicine, faculty, musculoskeletal pain, Saudi Arabia

## Abstract

We aimed to establish the local prevalence of musculoskeletal pain among faculty members in Saudi Arabia and describe the patient's risk factors and preventive measures that may reduce its burden.

An observational, quantitative, cross-sectional study was carried out to evaluate the prevalence of musculoskeletal pain and its risk factors among male faculty members in the College of Medicine and Dentistry, using a designed questionnaire based on the Standardized Nordic Musculoskeletal Questionnaire. Chi-square testing at a significance level of *P* < .05, was used for comparative analysis. SPSS version 26 was used for all analyses.

Ninety responders participated in the survey analysis. The prevalence of musculoskeletal pain among faculty members was 77.8%, and the most common site of musculoskeletal pain occurred at two different sites of the three (low back, neck, and shoulder), with a prevalence of 38.9%. As for risk factors of musculoskeletal pain, only age group showed a significant correlation with the site of musculoskeletal pain (*P* = .024), where patients in the younger age group (25–35 years old) were at higher risk of lower back pain, while participants in the older age group (36 to 44 years old and 45 years or older) were at higher risk of musculoskeletal pain in two different sites.

Musculoskeletal pain affects more than two-thirds of faculty members. In particular, low back pain is a common problem among faculty members. Age is a significant risk factor for the occurrence of musculoskeletal pain, with more than one site involvement in older age.

## 1 Introduction

Musculoskeletal pain linked to working conditions can be identified by multiple clinical manifestations in the bones, muscles, and joints, and is correlated with some habits or activities related to the patient's working environment.^[1]^ Although these symptoms could be disabling for some patients and reduce workers’ productivity, they can be easily prevented.^[2]^ Musculoskeletal pain threatens patients’ professional lives and reduces their overall quality of life. This, in turn, is negatively associated with health care costs for employees.^[3]^

It has been proposed that healthcare workers are most commonly affected by musculoskeletal pain.^[4]^ This could be attributed to its dual roles in clinical practice and academia. The prevalence of musculoskeletal pain among healthcare workers, including dentists, nurses, and physical therapists, is significantly high and can reach up to 97%, while neck and low back pain are the most common complaints.^[5–7]^ A study in Brazil showed a high prevalence of musculoskeletal pain among college professors (85.7%); the most common complaints were in the lower back (54.8%), followed by the neck (45.2%) and shoulders (23.8%).^[8]^ Another study revealed that among postgraduate students and faculty of dentistry in Spain, 79.8% had experienced musculoskeletal pain, and the neck was the most common area affected by 58%.^[9]^ Sirajudeen et al conducted the first study in Saudi Arabia, estimating the prevalence of work-related musculoskeletal disorderss among faculty members and reported musculoskeletal symptoms in more than half of the 55% reported musculoskeletal symptoms.^[10]^ However, the study population was confined to the faculty of the College of Applied Medical Sciences. Musculoskeletal pain in healthcare workers usually results from multiple risk factors, including an inconvenient working posture, a large volume of treated patients, administrative work, vibration, and repetition.^[6]^ A recent study has also demonstrated an increased incidence of pain with jobs that require increased physical activity from workers and exposure to risk factors related to their profession.^[11]^

Some of these risk factors have also been investigated. The most common were uncomfortable postures, carrying heavy materials, bending, or repositioning patients.^[12]^ However, these factors should consider the specialty within a profession, the years of experience, and the amount of work that a worker performs.^[13]^

The prevalence of musculoskeletal pain among faculty members is relatively high compared to other professions, although the accurate prevalence in gulf areas is still unclear.^[10]^ Hence, the present study aimed to establish the local prevalence of musculoskeletal pain among faculty members in Saudi Arabia and describe the patient's risk factors and preventive measures that may reduce its burden.

## 2 Materials and methods

### 2.1 Ethical approval and informed consent

Institutional research ethics board approval was acquired from our institution prior to conducting any study procedures. Participants’ consent was obtained for participation and publication of the study.

### 2.2 Study design

An observational quantitative cross-sectional investigation was conducted to evaluate the prevalence of musculoskeletal pain and its risk factors among faculty members in the College of Medicine and College of Dentistry at Prince Sattam bin Abdulaziz University, using a standardized Nordic musculoskeletal questionnaire.^[14]^ We evaluated the questionnaire's validity using a pilot test among five academic workers. After that, we made minor revisions and finalized the initial version of the questionnaire. A self-administered questionnaire was personally distributed to 105 participants, and their consent was obtained for participation and publication of the study. The inclusion criteria were as follows:

1. fully informed about the contents of the questionnaire and consented to participate in the study; and
2. full-time academic workers.

The exclusion criteria were as follows:

1. part-time academic workers
2. participants with incomplete questionnaire information.

### 2.3 Data collection

Data was collected through the responses to the questionnaire.

The questionnaire covered the following information:

### 2.4 Demographic information and baseline characteristics

College (either medicine or dentistry), gender, age group (25–35, 36–44, or ≥ 45 years), marital status, smoking status, height, weight, number of children, years of working in the academic field (< 5 years, 5–9 years, or ≥ 10 years), teaching hours per day (< 1 h, 1–2 h, 3–4 h, or ≥ 5 h), computer use in hours per day (< 1 h, 1–2 h, 3–4 h, or ≥ 5 h), position at work sitting or standing, sleeping hours per day (≤ 6 h, or 7–8 h). Body mass index (BMI) categories were designed according to WHO guidelines (BMI < 18.5, underweight: 18.5–24.99, normal: 25–29.99, overweight; ≥ 30, obese).^[15]^ The subject who never smoked or no longer smoking at the time of the survey was counted as “non-smoker”, and the subject who is still smoking as “smoker”.

#### 2.4.1 Musculoskeletal pain and work-related characteristics during the last 12 months

According to the anatomic site, musculoskeletal pain assessment including neck, shoulder, and low back; pain duration in days (0, 1–7 days, 8–30 days, > 30 days but not every day, or every day), change in work duties/ leisure activity due to the pain, seeking medical advice for the pain, and total length of time that the pain has prevented the participant from doing normal work (0, 1–7 days, 8–30 days, > 30 days but not every day, or every day. Survey questions were validated using Cronbach's alpha testing, with all questions showing correlations between 0.828 and 0.903 and a total α coefficient of 0.928.

### 2.5 Statistical analysis

All data were entered into a pre-designed and validated Excel sheet. Data are described in terms of frequencies and percentages for categorical variables. Musculoskeletal pain was classified into six groups: low back pain, neck pain, shoulder pain, musculoskeletal pain at two different sites of the three (low back, neck, and shoulder), musculoskeletal pain in all three sites (pain at all sites), or no pain at all. Comparative analysis was carried out for categorical variables using the Chi-square test at a significance level of *P* < .05. Data were analyzed using IBM SPSS (Statistical Package for the Social Science; IBM Corp, Armonk, NY, USA) to perform all statistical calculations, version 26 for Microsoft Windows.

## 3 Results

**Figure 1:**
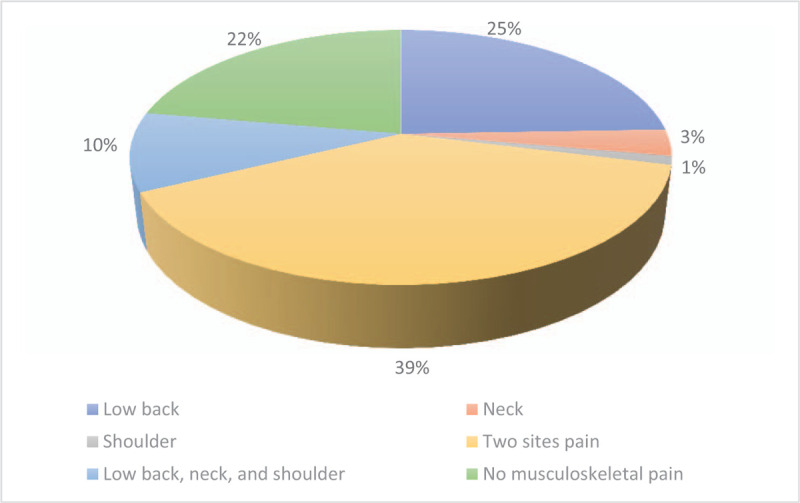
Prevalence of different sites of musculoskeletal pain.

Out of 105 distributed questionnaires, 90 male faculty members responded and returned the filled-up survey forms with a response rate of 85.7%. The prevalence of musculoskeletal pain among faculty members was 77.8%. The prevalence of musculoskeletal pain was also determined. The most common site of musculoskeletal pain among faculty members was pain occurring at two different sites, with a prevalence of 38.9%, followed by low back pain, with a prevalence of 24.4%. It is worth noting that healthy individuals represented 22.2% of the entire cohort, as shown in Figure 1. Socio-demographic data of the participants were explored based on the site of musculoskeletal pain that they had. It has been shown that participants from medical colleges were predominant in all groups. In addition, the age group 25 to 35 was more common in the lower back, and the healthy individuals, while the older age group ≥ 45 years was more common among the neck, shoulder pain groups, and pain in all three sites.

Years in the academic years, exercising, and BMI class varied among the six groups, while most of the participants were married and non-smokers. A description of the demographic data of the participants is shown in Table 1.

**Table 1:** Socio-demographic data of the included participants.

Socio-demographic data	Low back pain (%)	Neck pain (%)	Shoulder pain (%)	Two different sites pain (%)	Low back, neck, and shoulder pain (%)	None (%)
College						
Medicine	77.3	66.7	100.0	51.4	77.8	70.0
Dentistry	22.7	33.3		48.6	22.2	30.0
Age group (years)						
25–35	45.5	33.3		14.3	44.4	40.0
36–44	31.8			57.1		25.0
≥ 45	22.7	66.7	100	28.6	55.6	35.0
Working in academic field (years)						
< 5	45.5	33.3		22.9	44.4	20.0
5–9	31.8		100	40.0	33.3	50.0
≥ 10	22.7	66.7		37.1	22.2	30.0
Marital status						
Single	13.6	33.3		8.6		10.0
Married	86.4	66.7	100.0	91.4	100.0	90.0
Number of children						
0	22.7	33.3		11.4	11.1	25.0
1	22.7			25.7	11.1	15.0
2	22.7	66.7		17.1	22.2	35.0
3	18.2			22.9	22.2	15.0
≥ 4	13.6		100	22.9	33.3	10.0
Smoking						
Non-smoker	90.9	66.7	100	82.9	88.9	100
Smoker	9.1	33.3		17.1	11.1	
Exercise						
No	50.0	33.3	100.0	51.4	55.6	45.0
Yes	50.0	66.7		48.6	44.4	55.0
BMI^∗^ class						
Underweight						
Normal	13.6	33.3		22.9		10.0
Overweight	40.9	66.7		40.0	66.7	60.0
Obese	45.5		100.0	37.1	33.3	30.0

The social habits of the participants were also evaluated. All the groups had a higher percentage of participants with sleeping hours of 7–8 h/day, except for participants with neck pain and those with pain in all the three sites, they had ≤ 6 sleeping hours per day. Additionally, most group participants were more commonly working in the sitting position and teaching for more than five hours per day. However, the hours using computers varied between and 3–4 h and more than 5 h/day among the six groups, as shown in Table 2.

**Table 2:** Social and professional habits of participants.

Social and professional habits	Low back pain (%)	Neck pain (%)	Shoulder pain (%)	Two different sites pain (%)	Low back, neck, and shoulder pain (%)	None (%)
Sleeping per day (hours)						
≤ 6	36.4	66.7		40.0	66.7	40.0
7–8	63.6	33.3	100	60.0	33.3	60.0
Positioning						
Sitting	81.8	66.7	100	71.4	66.7	70.0
Standing	18.2	33.3		28.6	33.3	30.0
Teaching per day (hours)						
< 1	13.6			2.9		10.0
1–2	18.2			5.7	11.1	10.0
3–4	31.8			28.6	33.3	20.0
≥ 5	36.4	100	100	60.0	55.6	60.0
Computer use per day (hours)						
< 1	4.5	33.3		22.9		10.0
1–2	22.7	33.3		48.6	22.2	25.0
3–4	31.8	33.3	100	28.6	44.4	35.0
≥ 5	40.9	33.3		22.9	33.3	30.0

Based on the pain site, participants were asked a set of questions to evaluate their pain. It has been shown that 64.4% of the entire cohort had low back pain, either alone or combined with another pain at another site. Of the subjects who had low back pain, 60.3% had pain between 1 to 7 days during the past year, even though 53.8% performed their work even during pain. However, only 5.2% of the patients were hospitalized because of pain. While 12.1% had to change their duties because of pain, 32.1% reduced their work activity and 39.6% reduced their leisure activity due to pain. All responses are presented in Table 3.

**Table 3:** Responses of low back pain participants.

	Frequency	(%)
Have you ever had a low back problem (ache, pain, or discomfort)?		
No	32	35.5
Yes	58	64.4
Have you ever been hospitalized due to low back problem?		
No	55	94.8
Yes	3	5.2
Have you ever had to change jobs or duties due to low back trouble?		
No	51	87.9
Yes	7	12.1
What is the total duration that you have had low back trouble during the last 12 months?		
0	6	10.3
1–7 days	35	60.3
8–30 days	9	15.5
> 30 days but not every day	7	12.1
Every day	1	1.7
Has low back trouble caused you to reduce your activity during the last year?a. Work activity (at home or away from home)?		
No	36	67.9
Yes	17	32.1
b. Leisure activity?		
No	32	60.4
Yes	21	39.6
What is the total length of time that the low back problem has prevented you from doing your normal work (at home or away from home) during the last year?		
0	28	53.8
1–7 days	22	42.3
8–30 days	1	1.9
> 30 days but not every day	1	1.9
Have you been seen by a doctor, physiotherapist, chiropractor, or another such person because of low back trouble during the last 12 months?		
No	31	58.5
Yes	22	41.5
Have you had low back trouble at any time during the last seven days?		
No	42	79.2
Yes	11	20.8

Similarly, neck pain participants responded to another set of questions, which showed that 32.2% of the whole cohort had neck pain, either alone or combined with another pain at another site. Of the subjects who had neck pain, 7.9% had an accident that hurt their neck. In addition, 52.6% had pain between 1 and 7 days during the past year, despite 45.7% performing their work even during pain.

However, only 31.4% of patients with neck pain seek medical help, and 22.9% have neck pain during the past week. While 7.9% had to change their duties because of the pain, 28.6% reduced their work activity and 34.3% reduced their leisure activity due to pain. All responses are presented in Table 4.

**Table 4:** Responses of neck pain participants.

	Frequency	(%)
Have you ever had neck trouble (ache, pain, or discomfort)?		
No	52	57.7
Yes	38	32.2
Have you ever hurt your neck in an accident?		
No	35	92.1
Yes	3	7.9
Have you ever had to change jobs or duties because of neck trouble?		
No	35	92.1
Yes	3	7.9
What is the total length of time that you have had neck trouble during the last 12 months?		
0	1	2.6
1–7 days	20	52.6
8–30 days	14	36.8
> 30 days but not every day	3	7.9
Every day		
Has neck trouble caused you to reduce your activity during the last 12 months?a. Work activity (at home or away from home)?		
No	25	71.4
Yes	10	28.6
b. Leisure activity?		
No	23	65.7
Yes	12	34.3
What is the total length of time that neck trouble has prevented you from doing your normal work (at home or away from home) during the last 12 months?		
0	16	45.7
1–7 days	13	37.1
8–30 days	4	11.4
> 30 days but not every day	2	5.7
Have you been seen by a doctor, physiotherapist, chiropractor, or another such person because of neck trouble during the last 12 months?		
No	24	68.6
Yes	11	31.4
Have you had neck trouble at any time during the last seven days?		
No	27	77.1
Yes	8	22.9

As for participants with shoulder pain, 13.3% of the whole cohort had shoulder pain, either alone or in combination with another pain at another site. Of the subjects who experienced shoulder pain,

During the past year,31.3% had pain between 1 and 7 days, and 50% did not perform their job during the same duration. However, only 53.3% of patients with shoulder pain seek medical help, 33.3% had shoulder pain in the past 12 months, and 53.3% had pain during the past week. While 4.8% had to change their duties because of the pain, 46.7% reduced their work activity, and 53.3% reduced their leisure activity due to pain. All responses are presented in Table 5.

**Table 5:** Responses of shoulder pain participants.

	Frequency	(%)
Have you ever had shoulder trouble (ache, pain, or discomfort)?		
No	69	76.6
Yes	21	13.3
Have you ever hurt your shoulder in an accident?		
No	15	71.4
Yes, my right shoulder	3	14.3
Yes, my left shoulder	2	9.5
Yes, both shoulders	1	4.8
Have you ever had to change jobs or duties due to shoulder trouble?		
No	20	95.2
Yes	1	4.8
Have you had shoulder trouble during the last 12 months?		
No	7	33.3
Yes, my right shoulder	7	33.3
Yes, my left shoulder	5	23.8
Yes, both shoulders	2	9.5
What is the total length of time that you have had shoulder trouble during the last 12 months?		
0	1	6.3
1–7 days	5	31.3
8–30 days	4	25.0
> 30 days but not every day	4	25.0
Every day	2	12.5
Has shoulder trouble caused you to reduce your activity during the last 12 months?a. Work activity (at home or away from home)?		
No	8	53.3
Yes	7	46.7
b. Leisure activity?		
No	7	46.7
Yes	8	53.3
What is the total length of time that shoulder trouble has prevented you from doing your normal work (at home or away from home) during the last 12 months?		
0	4	28.6
1–7 days	7	50.0
8–30 days	2	14.3
> 30 days but not every day	1	7.1
Have you been seen by a doctor, physiotherapist, chiropractor, or another such person because of shoulder trouble during the last 12 months?		
No	7	46.7
Yes	8	53.3
Have you had shoulder trouble at any time during the last seven days?		
No	7	46.7
Yes	8	53.3

Different risk factors were compared across different pain sites using the chi-square test for categorical risk factors and one-way ANOVA for numerical risk factors at a significance level of *P* < .05. The risk factors were college, age group, years in the academic field, marital status, number of children, smoking, exercising, BMI class, sleeping hours, positioning, teaching hours, and computer hours, in addition to height, weight, and BMI.

Only the age group should have a significant correlation with the site of musculoskeletal pain (*P* = .024), where patients at a younger age (25–35 years old) were at a higher risk of having low back pain. In comparison, participants in the age group of 36 to 44 years old were at a higher risk of musculoskeletal pain in two different sites. In addition, subjects aged 45 years or older were more prone to pain at the two sites, as shown in Tables 6 and 7.

**Table 6:** Comparison of different risk factors over different sites of musculoskeletal pain using a chi-square test.

	Low back pain	Neck pain	Shoulder pain	Two different sites pain	Low back, neck, and shoulder pain	None	*P*-value
College							
Medicine	28.8%	3.4%	1.7%	30.5%	11.9%	23.7%	.334
Dentistry	16.1%	3.2%	0.0%	54.8%	6.5%	19.4%	
Age group (years)							
25–35	35.7%	3.6%	0.0%	17.9%	14.3%	28.6%	.024^∗^
36–44	21.9%	0.0%	0.0%	62.5%	0.0%	15.6%	
≥ 45	16.7%	6.7%	3.3%	33.3%	16.7%	23.3%	
Years in the academic field							
< 5	33.3%	0.0%	0.0%	33.3%	16.7%	16.7%	.766
5–9	23.1%	5.1%	2.6%	35.9%	7.7%	25.6%	
≤ 10	18.5%	3.7%	0.0%	48.1%	7.4%	22.2%	
Marital status							
Single	33.3%	11.1%	0.0%	33.3%	0.0%	22.2%	.649
Married	23.5%	2.5%	1.2%	39.5%	11.1%	22.2%	
Number of children							
0	31.3%	6.3%	0.0%	25.0%	6.3%	31.3%	.687
1	27.8%	0.0%	0.0%	50.0%	5.6%	16.7%	
2	22.7%	9.1%	0.0%	27.3%	9.1%	31.8%	
3	23.5%	0.0%	0.0%	47.1%	11.8%	17.6%	
≥ 4	17.6%	0.0%	5.9%	47.1%	17.6%	11.8%	
Smoking							
Non-smoker	25.0%	2.5%	1.3%	36.3%	10.0%	25.0%	.357
Smoker	20.0%	10.0%	0.0%	60.0%	10.0%	0.0%	
Exercise							
No	24.4%	2.2%	2.2%	40.0%	11.1%	20.0%	.892
Yes	24.4%	4.4%	0.0%	37.8%	8.9%	24.4%	
BMI^∗^ class							
Normal	21.4%	7.1%	0.0%	57.1%	0.0%	14.3%	.49
Overweight	20.9%	4.7%	0.0%	32.6%	14.0%	27.9%	
Obese	30.3%	0.0%	3.0%	39.4%	9.1%	18.2%	
Sleeping hours							
≤ 6 hours	21.1%	5.3%	0.0%	36.8%	15.8%	21.1%	.536
7–8 h	26.9%	1.9%	1.9%	40.4%	5.8%	23.1%	
Positioning							
Sitting	27.3%	3.0%	1.5%	37.9%	9.1%	21.2%	.898
Standing	16.7%	4.2%	0.0%	41.7%	12.5%	25.0%	
Teaching hours							
< 1 h	50.0%	0.0%	0.0%	16.7%	0.0%	33.3%	.760
1–2 h	44.4%	0.0%	0.0%	22.2%	11.1%	22.2%	
3–4 h	29.2%	0.0%	0.0%	41.7%	12.5%	16.7%	
≥ 5 hours	16.0%	6.0%	2.0%	42.0%	10.0%	24.0%	
Computer use hours							
< 1 h	33.3%	0.0%	0.0%	0.0%	0.0%	66.7%	.931
1–2 h	23.8%	4.8%	0.0%	38.1%	9.5%	23.8%	
3–4 h	18.9%	2.7%	2.7%	45.9%	10.8%	18.9%	
≥ 5 h	31.0%	3.4%	0.0%	34.5%	10.3%	20.7%	

**Table 7:** Comparison of different risk factors over different sites of musculoskeletal pain using the one-way ANOVA test.

	Mean	Standard deviation	*P*-value
Height (cm)			
Low back	170.45	9.465	.899
Neck	169.33	11.015	
Shoulder	176.00		
Two different sites pain	172.06	8.564	
Three different sites pain	172.78	10.509	
None	173.10	6.215	
Weight (kg)			
Low back	86.00	17.049	.656
Neck	73.00	21.703	
Shoulder	95.00		
Two different sites pain	84.20	14.644	
Three different sites pain	88.78	15.336	
None	90.00	24.688	
BMI^∗^ (kg/m^2^)			
Low back	29.5381	4.85776	.657
Neck	25.0484	5.16102	
Shoulder	30.6689		
Two different sites pain	28.3981	4.22885	
Three different sites pain	29.6481	3.75877	
None	29.9362	7.33227	

## 4 Discussion

Musculoskeletal pain limits patient activity, especially when jobs require certain positions for a long duration.^[16]^ Faculty members in medical schools are at a higher risk of musculoskeletal pain due to their work's nature, which requires long hours of sitting or standing without changing positions.^[17]^ However, the risk factors and prevalence of musculoskeletal pain among faculty members in Saudi Arabia remain controversial.

The present work explores the prevalence and risk factors of musculoskeletal pain among faculty members in Saudi Arabia. The study revealed that the most common musculoskeletal pain site among faculty members was pain occurring at two different sites of the three (low back, neck, and shoulder), with a prevalence of 38.9%, followed by low back pain (24.4%). In contrast, 22.2% of the patients were healthy.

Additionally, 64.4% of the whole cohort had low back pain, 32.2% had neck pain, and 13.3% of the whole cohort had shoulder pain, either alone or with other sites. Turning to risk factors of musculoskeletal pain, only the age group showed a significant correlation with the site of musculoskeletal pain (*P* = .024), where patients at a younger age (25–35 years old) were more at risk of having low back pain. In comparison, participants in the older age group 36 to 44 years old and 45 years or older were at a higher risk of musculoskeletal pain in two different sites.

Musculoskeletal pain was evaluated in different settings. Collins et al^[18]^ evaluated the prevalence and risk factors of musculoskeletal pain among employees in two academic bodies. The study included employees who spent more than 25% of their working hours in front of computers.

Among 852 participants, Collins et al^[18]^ demonstrated that the prevalence of neck pain was 58%, shoulder pain was 57%, and low back pain was 51%. Additionally, Collins et al^[18]^ did not find a significant difference in the site of pain with age group.

The present study illustrated that 64.4% of the whole cohort had low back pain, 32.2% of the whole cohort had neck pain, and 13.3% of the whole cohort had shoulder pain, either alone or with other sites. Furthermore, the age group was significantly correlated with the site of musculoskeletal pain (*P* = .024).

Abdulmonem et al^[19]^ examined the prevalence and risk factors of musculoskeletal pain in female teachers in Saudi Arabia through a survey analysis. Through 486 participants, Abdulmonem et al^[19]^ showed that low back pain was the most prevalent among the whole cohort. Additionally, Abdulmonem et al^[19]^ showed that the risk factors were BMI, teaching hours, and the presence of comorbidities.

Although the present study showed the highest prevalence of low back pain, only males from dentistry and medical colleges were recruited. Additionally, only age group was a significant risk factor for musculoskeletal pain, with BMI, teaching hours, and years working in academia as non-significant risk factors for pain, which could be attributed to the small sample size.

In addition, Dajpratham et al^[20]^ examined the prevalence of musculoskeletal pain among employees in dental schools in Thailand using a self-administered questionnaire. Dajpratham et al^[20]^ included 163 participants and showed that cervicobrachial pain was the most prevalent in the included cohort. Working in academia and clinical practice is the most common risk factor for pain.^[20]^

In the present study, neck pain was ranked second following low back pain. All recruited respondents were academic staff working in either medical or dental schools. Age was the most significant risk factor for musculoskeletal pain.

To our knowledge, this is the first study to establish the prevalence and risk factors of musculoskeletal pain among faculty members in Saudi Arabia. However, the present study had some limitations that may question the outcomes.

The study depended mainly on the responder's honesty and subjective opinion while responding to these questions to identify the prevalence and risk factors of the disease. In addition, the responders’ small sample size could have achieved statistical significance in the evaluation of risk factors. Furthermore, the study outcomes might have been affected by the influence of confounding factors on musculoskeletal pain.

## 5 Conclusion

Musculoskeletal pain, particularly low back pain, is a common problem among faculty members. Age is a significant risk factor for musculoskeletal pain, with more sites being involved in older age. These findings should be considered by decision makers in universities to improve the work environment for faculty members to reduce their pain and thus enhance their productivity. Future studies with larger sample sizes and objective assessments are needed to confirm the risk factors, in addition to preventive measures of skeletal muscle pain among faculty members in Saudi Arabia.

## Acknowledgment

This publication was supported by the Deanship of Scientific Research at Prince Sattam bin Abdulaziz University, Alkharj, Saudi Arabia.

## Author contributions

**Conceptualization:** Osama R. Aldhafian, Faisal A. Alsamari.

**Data curation:** Osama R. Aldhafian, Faisal A. Alsamari, Naif A. Alshahrani, Mohammed N. Alajmi, Abdulelah M. Alotaibi.

**Formal analysis:** Osama R. Aldhafian.

**Investigation:** Osama R. Aldhafian.

**Methodology:** Osama R. Aldhafian.

**Project administration:** Osama R. Aldhafian.

**Resources:** Osama R. Aldhafian.

**Software:** Osama R. Aldhafian.

**Supervision:** Osama R. Aldhafian.

**Validation:** Osama R. Aldhafian.

**Visualization:** Osama R. Aldhafian.

**Writing – original draft:** Osama R. Aldhafian, Faisal A. Alsamari.

**Writing – review & editing:** Osama R. Aldhafian, Faisal A. Alsamari, Naif A. Alshahrani, Mohammed N. Alajmi, Abdulelah M. Alotaibi, Naif Bin Nwihadh, Ayman K. Saleh.
